# From Agricultural Residues to Sustainable Boards: Complex Network Analysis of Binderless Composites

**DOI:** 10.3390/polym17223082

**Published:** 2025-11-20

**Authors:** Lucia Rossi, Luis A. Miccio, Emiliano M. Ciannamea, Pablo M. Stefani

**Affiliations:** 1 Instituto de Investigaciones en Ciencia y Tecnología de Materiales (INTEMA), Consejo Nacional de Investigaciones Científicas y Técnicas (CONICET), Universidad Nacional de Mar del Plata (UNMdP), Av. Colón 10850, Mar del Plata B7600FDQ, Argentina; luciarossig@gmail.com (L.R.); emiliano@fi.mdp.edu.ar (E.M.C.); pmstefan@fi.mdp.edu.ar (P.M.S.); 2 Departamento Polímeros y Materiales Avanzados: Física, Química y Tecnología, University of the Basque Country (UPV/EHU), P. Manuel Lardizábal 3, 20018 San Sebastián, Spain

**Keywords:** particleboard, lignocellulosic materials, biobased adhesives, agroindustrial wastes, self-bonded, brewer spent grain, complex network approach, biomass pretreatments

## Abstract

The transition toward sustainable panel technologies is driving intensive research on binderless boards and self-bonded lignocellulosic composites. Particleboard, an engineered wood composite made by hot pressing wood particles with synthetic adhesives, is among the most widely produced wood-based panels due to cost-effectiveness and versatility. However, pressure on forest-derived raw materials and concern over formaldehyde emissions are accelerating the search for renewable resources and greener routes. Residues and underutilized materials from agro-industrial, food, and forestry sectors (such as cereal straws, sugarcane bagasse, brewer’s spent grain, and fruit-processing by-products) offer a sustainable alternative, enabling waste valorization, lowering environmental burdens, and supporting circular bioeconomy models. Binderless boards, produced without adhesives, exploit natural bonding among lignocellulosic components, including lignin softening, thermoplasticization, and covalent crosslinking during hot pressing. This review adopts a complex network approach to systematically map and analyze the scientific landscape of binderless board production. Using citation-based networks from curated seed papers and their first- and second-degree neighbors, we identify thematic clusters, with cluster “A” as the research core. The examination of this cluster, complemented by word-cloud analysis of titles and abstracts, highlights prevalent raw materials and key research lines, like raw-material sources and lignocellulosic composition, processing parameters, and pretreatment strategies. Based on these findings, brewer’s spent grain is selected as a representative case study for cost analysis. This approach synthesizes the state of the art and reveals emerging directions, research gaps, and influential works, providing a data-driven foundation for advancing self-bonded lignocellulosic composites.

## 1. Introduction

Particleboards are engineered wood-based composite panels manufactured from wood particles bonded with synthetic adhesives and hot-pressed into sheets. Wood raw materials for particleboards include logs (i.e., Eucalyptus grandis), sawmill residues (chips, sawdust), and, in some countries, recycled wood, whereas urea–formaldehyde (UF) resins are more commonly used as synthetic adhesives, although phenol–formaldehyde (PF) and melamine–formaldehyde (MF) resins can be used to improve moisture resistance. In modern continuous production lines, milling, drying, and screening ensure proper particle size. The board typically consists of a low-density core of coarse particles and higher-density surface layers of finer particles, providing a sandwich-like structure with an advantageous stiffness-to-weight ratio under bending loads [1].

**Figure 1:**
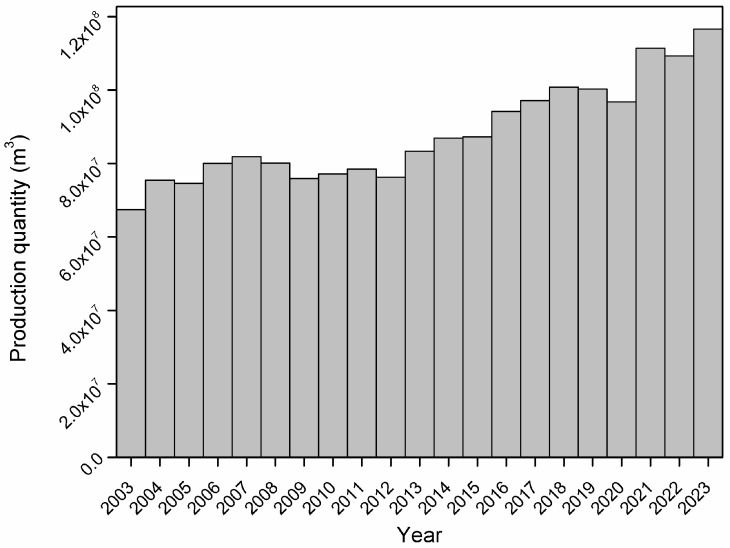
Production quantity of particleboards worldwide [4].

Owing to their relatively low cost, versatility, and adequate performance, wood-based particleboards have become one of the most widely used engineered wood products, particularly in the furniture industry, interior construction, and housing sectors [2,3]. The scale of this industry is considerable: global production of particleboards has exceeded 116 million m^3^ in recent years, with a market value of approximately USD 25.1 billion in 2023 [4]; see Figure 1.

Particleboards originated from the need to utilize large quantities of sawmills residues. From a sustainability perspective, they enhance circular economy principles by converting wood residues and by-products into durable engineered materials. However, the growth of particleboard market is accompanied by increasing pressure on natural wood resources. Rising demand for timber coincides with competing land uses for food and bioenergy crops, while deforestation and the limited pace of afforestation threaten the long-term availability of raw materials. Several studies have highlighted that the renewal cycles of forest plantations are not sufficient to meet industrial needs, creating risks of resource scarcity and price volatility [5,6]. These environmental and economic challenges have driven a search for alternative raw materials and greener production technologies, setting the stage for the exploration of agro-industrial residues as sustainable feedstocks.

As a response to these concerns, the substitution of wood with lignocellulosic fibers and agricultural residues has become a growing area of research [7,8]. A particularly promising strategy is the use of agricultural and agro-industrial by-products, which are available in large volumes and often pose disposal challenges due to their high organic load. Examples include cereal straws, sugarcane bagasse, oil palm residues, rice husks, grapevine pruning, brewer’s spent grain (BSG), and fruit-processing residues, among many others [9]. According to FAO and recent reviews, these residues represent millions of tons of underutilized biomass annually, and their valorization into engineered materials offers both environmental and economic benefits. In Argentina, where the agro-industrial sector is a key pillar of the economy, several residues have been identified as particularly suitable for the production of binderless boards. These include sugarcane bagasse, wheat and corn straws, BSG from the beer industry, and fruit residues from grapes and olives [10,11,12]. Mapping studies highlight additional promising sources such as residues from bioethanol production (maize), viticulture by-products, and forestry residues from native species, especially in provinces like Córdoba, La Rioja, and La Pampa [10]. The large-scale availability of these feedstocks opens the possibility of reducing the environmental burden of waste disposal (e.g., open burning, landfilling) while supporting circular economy models through the development of cost-effective raw materials and local bio-based value chains. Substituting wood with such residues not only conserves native forests and mitigates deforestation but also contributes to reducing greenhouse gas emissions linked to agricultural waste management.

From a global perspective, the utilization of agro-residues in particleboard production addresses broader sustainability goals by reducing pressure on forests, promoting efficient use of biomass, and creating socio-economic opportunities in rural regions [7,13,14]. International regulations and initiatives aimed at forest preservation and sustainable land use further reinforce the urgency of developing alternative raw materials for wood-based industries [4].

Research has shown that agricultural residues can be incorporated into board manufacture using either synthetic adhesives (for example, urea–formaldehyde, melamine–urea–formaldehyde, or polymeric MDI) or bio-based adhesives derived from proteins, lignin, starch, or tannins [7,9]. There are numerous examples of boards based on cornstarch [15], olive stones [16], sorghum bagasse [17], oil palm residues [18], rice husks [19], hemp [20], plum pruning [21], tobacco stems [22], coconut husks and rice husks [23], sunflower and flax shives [24], BSG and wood [25], and sugarcane bagasse combined with bamboo [26]. While the global wood adhesive market is projected to reach USD 21.8 billion by 2028 [27], increasing awareness of the risks posed by formaldehyde-based resins, including carcinogenicity and VOC emissions, has accelerated the search for sustainable adhesive systems [28,29,30]. In this context, soy protein, lignin-, and starch-based adhesives have received renewed attention [14].

An even more radical approach is the manufacture of boards without any added adhesives (the so-called binderless boards). This technology exploits the self-bonding capacity of lignocellulosic materials, where lignin and hemicelluloses soften, plasticize, and undergo condensation reactions during hot pressing, resulting in sufficient interparticle bonding to produce panels with competitive properties [6,31,32]. Numerous studies have demonstrated the feasibility of binderless boards from a wide array of residues, including rice husk [33], rice straw [34,35], wheat straw [20,36,37], sugarcane bagasse [38,39], miscanthus [40], BSG [41], vineyard pruning [42], wheat bran and banana peels [43], oil palm trunk [5,18,44,45], coconut husk [46,47,48,49], bamboo [50,51,52], coriander [53], kenaf [38,54,55,56], coffee husk [57,58], and hemp [6,20,59,60].

Commercial specifications can be achieved in binderless boards by optimizing pressing conditions (time, temperature, pressure) and by applying pretreatments to improve fiber bonding [32,61,62,63,64]. While the exact mechanism of interparticle bonding is still under debate, it is generally attributed to a combination of mechanical contact, molecular interdiffusion, chemical bonding, and structural rearrangements [49,65,66,67]. In particular, the polyphenolic structure of lignin plays a central role: at temperatures near its glass transition, lignin becomes mobile, enabling interparticle adhesion through both covalent linkages and secondary interactions [49,68]. Studies have confirmed the formation of covalent bonds (including lignin–furfural and lignin–polysaccharide cross-links) that provide mechanical strength comparable to that of conventional resin-bonded panels [65,69,70].

The rapid growth of research on sustainable particleboard technologies has generated a vast and fragmented body of literature, making it challenging to obtain a clear overview of the field. Therefore, in order to frame our studies more accurately, we employed a complex citation network approach, which allows us to map the scientific landscape and identify the main thematic areas driving innovation in binderless composites and closely related topics. Starting from carefully selected seed papers and expanding through first- and second-degree citation neighbors, we access a representation of the knowledge map where papers are represented as nodes and citations as edges. By applying community detection and word-cloud analyses, we uncover distinct clusters of research activity (see Figure 1). Among them, the cluster on binderless fiberboards and self-bonded lignocellulosic composites emerges as a central hub, interlinked with related areas such as biomass pretreatments, bio-based adhesives, and thermal modification strategies. This network-based perspective not only synthesizes the state of the art but also highlights influential works, research gaps, and emerging directions that traditional narrative reviews may overlook.

**Figure 2:**
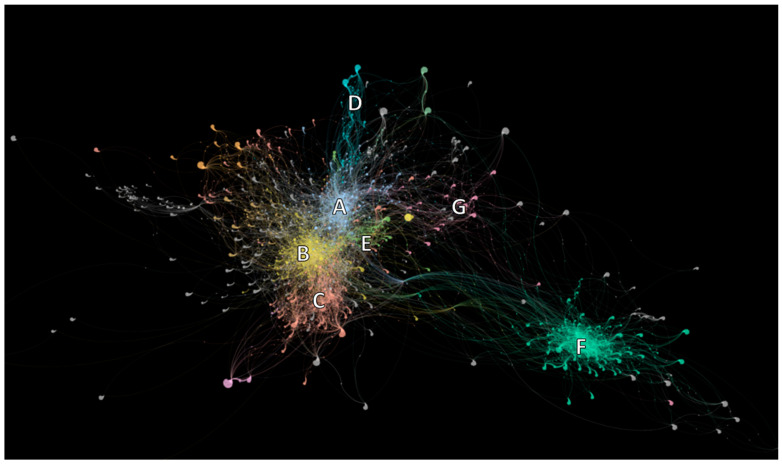
Knowledge map of the scientific landscape on sustainable particleboard technologies obtained through complex citation network analysis. The network was constructed from seed papers and their first- and second-degree citation neighbors, with nodes representing individual publications and edges representing citation links. Community detection reveals distinct thematic clusters. This structure provides a data-driven knowledge map, highlighting the interconnections between clusters and framing the research gaps and emerging directions in binderless board development.

Thus, the development of binderless fiberboards, particleboards, and self-bonded composites represents not only a sustainable use of underutilized residues but also an opportunity to fundamentally rethink board production. This line of research, which emerges prominently in cluster “A” of our citation network analysis (see Figure 2), reflects a shift towards bio-based, circular, and adhesive-free panel technologies that may reshape the future of the industry.

## 2. Methods

The complex network-based methodology employed in this review provides a structured analysis of the scientific landscape surrounding binderless boards and self-bonded lignocellulosic composites. Even when focusing on such a relatively narrow research area, the number of publications has grown rapidly and is highly fragmented across materials science, forestry, bioenergy, and waste valorization domains. This makes it impossible to exhaustively review the literature using traditional methods. In this context, informatics tools such as complex citation networks and ranking of the most influential works offer valuable instruments to systematize knowledge and to uncover emergent themes that might otherwise remain hidden [71]. A complex network of scientific publications was constructed by selecting seed articles (see Table S1) representative of binderless boards, bio-based adhesives, and agro-industrial residue utilization. Using their Digital Object Identifiers (DOIs), metadata and citation information from crossref.org was extracted.

**Figure 3:**
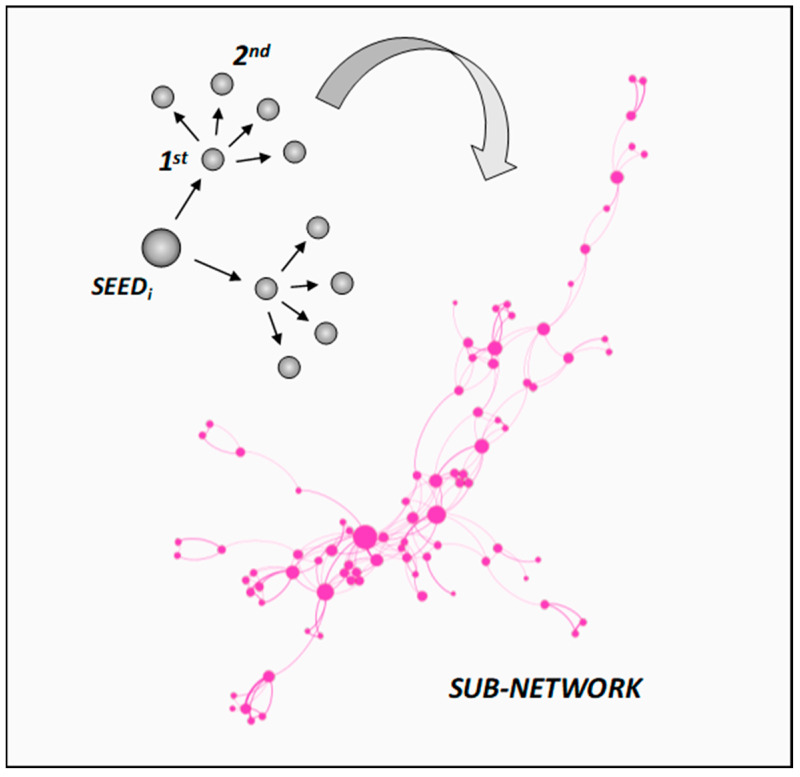
Schematic representation of each seed subnetwork construction.

Each seed was expanded through its first-degree (direct references) and second-degree neighbors (references of references), thereby ensuring that both foundational and emerging works were incorporated into the seed-related network (see Figure 3).

These networks were then merged into a unified network (duplicates were removed to avoid redundancy), resulting in a consolidated network with over 13,000 nodes and nearly 20,000 edges. PageRank was used to find influential works (as shown in Figure S1, it identifies those that are consistently cited across the network, signaling their long-term influence).

The workflow was implemented in Python 3.10, with custom scripts for citation retrieval and data curation. Clustering analysis was performed in Gephi 0.9 using the modularity optimization algorithm, also known as the Louvain or fast-unfolding method (see Figure S2). This algorithm seeks to maximize the modularity score, which quantifies the density of links within clusters compared to links between clusters.

**Figure 4:**
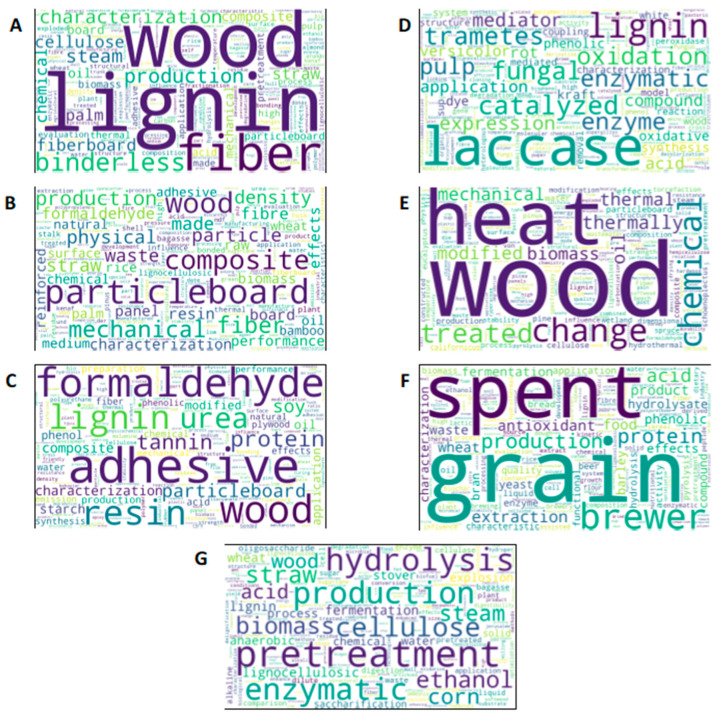
Word clouds of the main research clusters (**A**–**G**) identified in the citation network. Each word-cloud was generated from the titles of the papers contained within a given cluster. Word size corresponds to the relative frequency of occurrence across titles, with larger words indicating more prominent and recurrent concepts.

Word clouds were generated to visualize the dominant concepts and recurring terms within each thematic area. This methodology provides a simple but powerful tool to interpret the structure of the citation network. Since each cluster groups papers that are closely connected through citations, their titles collectively encode the dominant research focus of that community. Counting the frequency of terms in these titles allows us to identify which concepts are most characteristic of the cluster. High-frequency terms reflect recurring themes, experimental approaches, or materials that are central to the body of work, while less frequent terms indicate more peripheral topics. The resulting word clouds translate this statistical information into a visual format where word size directly represents importance. This offers an intuitive way to grasp the thematic orientation of each cluster, validate the coherence of the network partition, and compare the relative emphasis of different research communities. For example, for cluster “A” in Figure 1, words such as lignin, pretreatment, pressing temperature, and agro-residues emerged as central (see Figure 4). This combination of network metrics and text mining helped us to objectively characterize the research fronts and knowledge gaps across the field. A more detailed description of the clusters is presented in Section S2 of the Supplementary Information File.

## 3. Chemical Composition of Lignocellulosic Raw Materials

**Figure 5:**
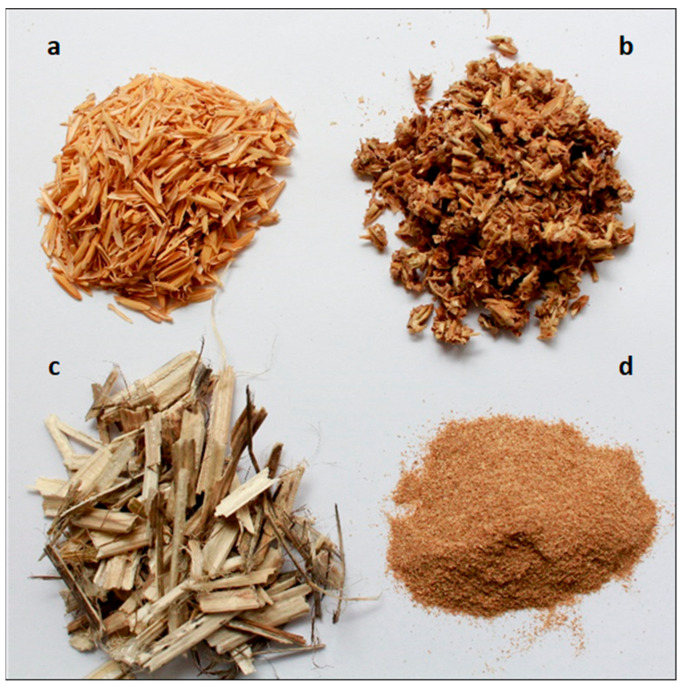
(**a**) Rice husk, (**b**) BSG, (**c**) hemp, and (**d**) grinded BSG (own photo).

The selection of raw materials for the fabrication of binderless boards is a critical factor, as their chemical composition substantially influences the final performance of the boards. Wood-based materials typically exhibit high cellulose and lignin content with low hemicellulose, favoring dimensional stability, while non-wood feedstocks (e.g., agricultural residues) often have elevated hemicellulose levels, which can compromise water resistance. Usually, the agro-industrial lignocellulosic main components fluctuated in a range of 20–50% cellulose, 25–40% hemicellulose, and 15–30% lignin [7,31]. Table 1 presents some of the raw materials used in various studies for the preparation of binderless boards, along with their main chemical composition: cellulose, hemicelluloses, lignin, ash, and extractives. Figure 5 shows some of the raw materials used for binderless board production (rice husk, BSG and hemp).

Understanding the interactions among cellulose, hemicellulose, and lignin is fundamental to elucidate binderless board engineering and overcome the inherent limitations associated with each individual component [31,68,72]. Hemicelluloses serve as primary contributors to moisture absorption and biological degradation in boards due to their hydrophilic nature. Reducing the hemicellulose content enhances water resistance by minimizing the abundance of hydroxyl groups that attract water molecules. However, this reduction often compromises inter-fiber bonding strength, as hemicelluloses play a vital role in facilitating hydrogen bonding and mechanical interlocking between fibers [72,73].

Lignin, an aromatic polymer with hydroxyl, carbonyl, and carboxylic functional groups, acts as a natural adhesive by forming hydrogen bonds and covalent crosslinks during hot pressing. Its presence at fiber surfaces improves interfiber adhesion through thermally activated self-bonding mechanisms. Moroever, mechanical properties of particleboards and moisture susceptibility of non-wood feedstock can be mitigated by extractives or lignin-rich additives [52]. For example, adding exogenous lignin or black liquor improves mechanical properties by enhancing crosslinking [32,33]. However, lignin is also responsible for ultraviolet degradation, limiting outdoor durability [32,64]. Extractives may counteract hemicellulose-related instability but can evaporate during hot pressing, causing delamination [74,75].

Optimizing processing conditions (e.g., pressure, temperature) remains critical to balance bonding efficiency and material integrity [31,32,62]. These conditions must be adapted to the specific chemical composition of each lignocellulosic source to promote self-bonding without inducing excessive thermal degradation. A deeper understanding of the interactions among cellulose, hemicellulose, lignin, and minor constituents is therefore essential for harnessing the intrinsic adhesive potential of lignin while mitigating the instability associated with hemicelluloses. This balance ultimately governs the mechanical performance and durability of binderless boards.

**Table 1:** Chemical composition of lignocellulosic raw materials to produce binderless from literature.

Raw Material	wt% Dry Sample			
	Cellulose	Hemicellulose	Lignin	Ash
Arundo donax [76]	29.2–43.1	14.5–32	19.2–24.3	4.2–6.1
Bagasse pith [38,39,69,77,78,79]	30.9–56.9	28.4–29.2	16.6–21.7	2.4–5.1
Bagasse rind [38]	31.1	22.6	20.8	-
Bamboo [50,51,52]	35.2–65	17–29.3	18–27.6	-
Banana bunch [80]	51.1	12.8	14.8	12.4
Barley straw [81]	34.8	27.9	14.6	5.7
Brewer’s Spent Grains [41]	9.5	28.7	11.2	3.3
Camphor tree [64]	43	25.1	22.7	0.1
Castor seed [82]	18.3	3.8	30.8	7.5
Chestnut [83]	51.9 (^2^)	-	25.2	-
Coconut coir [47,49]	21.1–37	8.5–23	29.2–42	1.8–2.5
Coriander [53]	34.7	36.9	1.0	-
Corn cobs–stalk biomass [34]	43.2	31.8	14.6–16	2.4–3.2
Empty fruit bunches [84]	64.4–67 (^2^)		20.9–24.5	-
Eucalyptus wood [85]	43.4	22.3	31.7	-
Flax fiber [86,87]	61–71	16–20.6	1.8–5.7	-
Flax shiv [86]	39.9–41	25.2–26.8	23–30.3	-
Gelam bark wood [83]	37.6	41.2	47.7	1.2
Hemp fiber [86]	60–76.2	12.8–22.4	3.7–5.7	-
Hemp shiv [60,86,88]	34–60	15–37	15–30	1.6
Kenaf powder [54]	84 (^2^)	-	25.3	-
Miscanthus [40,89]	42.6	21.1	19.9	0.7
Oak wood [24]	41	26	26	-
Oil palm (^1^) [5,90]	1.0–52.2	1.6–58.9	17.2–27.4	2.2–2.9
Rape straw [67]	40	-	19–21	-
Rattan waste [91]	34.3	45.6	21	5.4
Rice husk [33]	31.3	18.1	28.2	17.1
Rice straw [34,35]	30–36.5	25–30	8.9–12.3	13.3
Rye straw [81]	37.9	32.8	17.6	3
Softwood [35]	42–46	7–12	25–28	0.3–1.3
Sunflower bark [24]	43–48	19.5–23	21–32	-
Wheat straw [37,52]	39.7	30.6	17.7	7.7
White pine [83]	40.3 (^2^)		50	1

(^1^) Trunk, bark, leaves, and frond; (^2^) holocellulose.

## 4. Pretreatments Strategies Applied to Lignocellulosic Raw Materials

This section explores some of the most commonly used pretreatments applied to raw materials in the fabrication of binderless boards. These pretreatments could modify the chemical composition of the materials, as well as affect adhesion, interparticle bonding quality, and water resistance. As observed in the network analysis, previous research has studied the use of pretreatment methods. Group “G” highlighted the physical, chemical, physicochemical, and biological pretreatment applied to improve boards performance. Most studies focused on the mechanical pretreatment, that is, reducing particle size by cutting or milling the materials [31,32]. By increasing the surface area, the interaction between particles or fibers is enhanced. In some studies, the use of thermal and hydrothermal treatments, such as steam injection and steam explosion, resulted in boards that met recognized industrial standards, due to the development of more reactive sites in the material and its contribution to self-bonding. Other studies have explored chemical modification by acidic or alkaline treatment [91,92], Fenton’s reagent [31,84], enzymatic pretreatment (laccase oxidation) [52,93], and fungal degradation [31,94], achieving improvements in the properties of binderless boards due to the generation of free radicals on the particle surface. In general, lignocellulosic biomass undergoes additional processing after milling; however, in some instances, high-quality binderless fiberboards can be produced without further treatment [31,37,65,73]. This study focuses on mechanical methods, thermal techniques (steam explosion and thermomechanical treatment), chemical approaches, and biological processes (microbial and enzymatic).

### 4.1. Mechanical Treatments (Milling, Grinding, Refining)

The particle size and shape of the raw materials (fibrous or granular) have been shown to influence the mechanical properties and water absorption due to their effect on the bonding mechanisms between particles [31,32,60]. Milling, cutting, or grinding are common pretreatments for fibers and particles. In previous studies, the reduction in particle size has been shown to improve the quality of the boards, increasing the IB strength and/or MOR, since the small particles enlarge the contact surface area and enhance the self-bonding between particles [42,61,73]. This occurs because finer particles penetrate voids and pores of the material structure, compact more effectively with fewer overlaps, and form more uniform and denser structures, resulting in a more homogeneous board [66,95]. Consequently, binderless boards made from smaller particles exhibit improved dimensional stability and smoother surfaces compared to those from larger particles. In line with that, Ahmad et al. (2019) reported that rattan particles with an average size of 50 µm achieved the highest MOE, MOR, and IB values, while binderless boards made from 500 µm particles showed lower mechanical properties [95]. Mawardi et al. (2022) demonstrated that, in oil palm wood binderless boards, particle size influences both flexural strength and water resistance, with an optimal size of around 70 µm [18].

The WA ability of the panels depends on the particle size; larger particles show a higher percentage of WA, absorbing more water than smaller sizes [42,96]. Some studies on binderless boards from oil palm trunks reported a reduction of almost 40% in WA when using smaller particles (70 µm) [18]. Ahmad et al. (2019) could not measure WA and TS for boards made from 500 µm particles due to them breaking into small pieces after they were extracted from water, obtaining better values for boards made from particles of 50 µm [95]. Ferrández-Villena et al. (2022) produced binderless boards from vineyard prunings and found that particles smaller than 250 µm resulted in lower WA and TS values compared to boards made from particles in the 1000–2000 µm range [42].

For fiberboard production, grinding promotes fiber separation and increases surface area, enhancing inter-fiber bonding during hot pressing [31,97]. Lee et al. (2006) reported that increasing fibers’ slenderness ratios (L/D) from 3 to 26 improved MOE, MOR, and IB, whereas excessive refining led to property loss due to fiber damage [97]. However, the reduction in particle size involves additional energy and time costs that must be taken into consideration during the production process [32].

### 4.2. Thermal and Hydrothermal (Steam Explosion, Steam Injection)

Thermal pretreatments have been extensively studied for their ability to improve the performance of adhesive-free lignocellulosic boards. This improvement was notably observed in the analysis of group “E”, highlighting the role of thermal treatments in several studies focused on producing composites from oil palm, sugarcane bagasse, eucalyptus, and recycled pine.

Hydrothermal pretreatment involves the use of water vapor. In this context, steam explosion pretreatment is applied prior to board molding, whereas steam injection is applied during the hot-pressing stage. This method requires a steam atmosphere during pressing, using equipment in which the mold is enclosed within a pressurized chamber that allows the injection of heated steam. The steam explosion method partially hydrolyzes hemicelluloses into sugars via auto-hydrolysis, while simultaneously exposing cellulose and promoting lignin condensation [80,84,98]. During this process, the lignin content proportionally increases and redistributes onto the cellulose surface, enhancing hydrophobicity and improving the IB strength of binderless boards [61,73,99,100]. Audibert et al. (2025) reported an increasing IB value, from 0.22 MPa to 0.65 MPa, with an increment of temperature treatment from 197.5 °C to 207.5 °C [100]. The rapid decompression at the end of the residence time causes the lignocellulosic material to physically and chemically explode, breaking down its structure into smaller fractions. This results in hemicellulose degradation and lignin softening, which coats the fiber surfaces and acts as a self-adhesive component [20,73]. Table 2 shows the positive effects of steam explosion pretreatment on binderless board formation, and the improvement in mechanical properties has been demonstrated for various lignocellulosic materials, such as *Arundo donax* L. [101], banana bunch [80], coconut husk [48,102], hemp shives and wheat straw [20], rice straw [61], and oil palm wastes [84,90].

Steam explosion pretreatment has been shown to significantly enhance the mechanical properties of binderless boards, enabling them to meet several international standards. For instance, binderless fiberboards produced from steam-exploded rice straw were shown to meet the Japanese Industrial Standard A 5905-2003 [104] for IB exceeding the typical threshold of 0.3 MPa [61,103]. Similarly, binderless boards from steam-exploded wheat straw achieved a MOR around 15 MPa, fulfilling the standard EN 312 P3 [105] value requirements. Additionally, most of these boards made from hemp or wheat straw exhibited MOE values over 1900 MPa, meting EN 312 P3 standards [20].

The achievement of such standards illustrates the potential of steam explosion as a viable pretreatment method for producing high-quality, adhesive-free lignocellulosic composites.

### 4.3. Chemical Pre-Treatments (Acid Hydrolysis, Alkaline Treatment, Oxidation Agents)

Acid pretreatments are widely used to hydrolyze lignocellulosic components in biomass, primarily targeting the removal of hemicellulose and a small fraction of lignin. This process breaks the van der Waals forces, hydrogen bonds, and covalent bonds that maintain the structural integrity of the biomass, facilitating hemicellulose solubilization and enhancing fiber exposure [29,31,106]. Acid pretreatments can be performed using either concentrated or dilute acids, with the most commonly used being sulfuric, nitric, acetic, and hydrochloric acids.

Studies have shown that nitric acid can create strong bonds for auto-adhesion of lignocellulosic materials surface, as demonstrated in the treatment of maple panels. During nitric acid pretreatment lignin is primarily oxidized, nitrated, and hydrolyzed at room temperature. Subsequently polysaccharides are oxidized and hydrolyzed, and additional alteration of lignin takes place at temperatures above 100 °C [29,107].

The use of organic acids, including acetic acid and sulfuric acid, for pretreatment of wood fibers has been reported. Acetic acid pretreatment resulted in less cellulose degradation than sulfuric acid, due to its solvent effect on lignin. Acetic acid also achieved higher hemicellulose removal and better pulp yield with higher cellulose content, making it a promising method for extensive biomass utilization while alleviating cellulose damage compared to sulfuric acid [106].

Dilute acid pretreatment has demonstrated significant benefits for binderless board production by partially decomposing hemicellulose, removing ash content, and reducing surface rigidity, which improves fiber compressibility during hot pressing [31,108]. This treatment also exposes functional groups on cellulose surfaces, thereby enhancing chemical bonding and self-adhesive properties in the resulting boards. For instance, studies with dilute acid-pretreated wheat straw have shown improved tensile strength and better mechanical performance upon hot pressing [108]. Combining acid pretreatment with other methods, such as steam explosion, can further improve WA and TS, leading to more durable binderless boards suitable for various industrial applications.

Alkaline pretreatment has been reported to improve internal bond strength and dimensional stability in some binderless boards, especially when combined with optimized hot-pressing conditions. The common reagents used in alkaline pretreatment are sodium hydroxide (NaOH), calcium hydroxide (Ca(OH)_2_), potassium hydroxide (KOH), aqueous ammonia (NH_4_OH), and oxidative alkali. Sodium hydroxide has been shown to be effective in increasing MOR and IB of binderless particleboards from rattan furniture waste, improving digestibility and hydrolyzing hemicellulose, and enhancing dimensional stability [31,91,109]. However, alkaline treatment can also cause cellulose degradation and morphological changes in fibers, such as fibrillation and crack formation, which may affect mechanical properties if not properly optimized [31,36,110]. Studies have shown that alkaline-treated fibers may exhibit increased WA due to a higher content of exposed hydrophilic groups [92].

Pretreatment using Fenton’s reagent, composed of ferrous chloride and hydrogen peroxide, is an advanced oxidation process that has demonstrated significant potential for activating lignocellulosic fibers used in binderless board production. Fenton’s reagent activates fiber components that covalently bond with low-molecular-weight degraded compounds, contributing to self-bonding in binderless fiberboards [29,32]. This oxidative treatment facilitates fiber surface activation by generating highly reactive hydroxyl radicals capable of breaking down complex lignin structures and modifying fiber surface chemistry. The Fenton process enhances adhesive bonding between fibers or particles by increasing surface reactivity, which helps improve the mechanical integrity of boards produced without synthetic adhesives [29,31,111].

Milawarni (2018) further investigated the optimization of Fenton pretreatment conditions using different compositions of hydrogen peroxide and ferrous sulfate as catalysts [58]. Their work showed that this combined chemical oxidation, when followed by thermal treatment, enabled the production of binderless boards with mechanical properties comparable to or exceeding those of boards manufactured with commercial adhesives. The oxidation targets lignin components within the raw material, effectively altering its structure and promoting self-bonding during hot pressing. This approach offers an environmentally friendly alternative to synthetic adhesives, leveraging oxidative activation to improve fiber–fiber interactions and board stability. Overall, Fenton oxidation pretreatment provides a promising chemical modification strategy to enhance the performance of binderless boards, particularly by improving fiber surface characteristics and lignin reactivity. Further work continues to optimize reagent ratios and treatment parameters to balance fiber activation with process sustainability for industrial applications in sustainable composite material production.

### 4.4. Biological Pre-Treatments (Fungal and Enzymatic Laccase)

Laccases and laccase–mediator systems emerged as prominent keywords in cluster “D”. The research network emphasizes these pretreatments for enhancing the adhesion properties of lignocellulosic raw materials and improving the production of self-bonded boards. Some studies have reported the use of enzymatic pretreatment of lignocellulosic materials to modify their surface structure [31,32,93]. They have the advantage of being environmentally friendly and requiring lower disposal costs, less energy consumption, and milder processing conditions than other pretreatment strategies.

Laccase treatments have been demonstrated to oxidize lignin and promote crosslinking reactions on fiber surfaces, thereby enhancing the tensile strength of binderless boards. For example, treatments combining laccase enzymes with xylanase improved adhesion in wheat and rapeseed straw fibers [52]. Nasir et al. (2013) optimized the conditions for laccase pretreatment and hot pressing in binderless board production from wood fibers [93]. The laccase oxidized lignin into stable phenoxy radicals, which acted as crosslinking agents, creating a thin, uniform, plasticized lignin layer on fiber surfaces that significantly improved self-bonding without the need for synthetic adhesives. In addition to improving mechanical properties, enzymatic pretreatments with laccase increase water resistance and promote environmentally friendly processing through surface functionalization and lignin modification.

Biologically pretreated bamboo green residues were reported to increase board density upon fermentation. However, a slight decrease in MOR and MOE was observed, attributed to cellulose degradation during fermentation [51].

## 5. Effects of Processing Parameters on Board Properties

Key processing parameters during hot pressing include pressing temperature, duration, and applied pressure. Elevated temperatures soften the lignin in the pretreated biomass, allowing it to act as a natural adhesive that coats and bonds fibers. Moisture evaporation during pressing can further promote the formation of covalent bonds among the chemical components of lignocellulosic biomass, enhancing board cohesion. Temperature notably influences the mechanical properties of binderless fiberboards, particularly in the absence of synthetic adhesives. Along with temperature, pressing time and pressure significantly affect board performance [18,33,112,113].

These processing conditions induce chemical and physical changes in the lignocellulosic material that improve adhesion and mechanical strength up to an optimal point. For instance, Wang (2022) observed that pressing rice husk at 160 °C caused degradation and extraction of hemicellulose and lignin from interfibrillar regions, enhancing bonding within the board matrix and resulting in improved MOR [33]. However, some studies have reported that excessively high temperatures may cause over-degradation, leading to a decline in mechanical properties [41,46,48]. Therefore, optimizing temperature, time, and pressure is crucial to activate lignin effectively while minimizing thermal damage, thereby maximizing board quality.

Controlling moisture content is a critical factor in the processing of binderless boards, as it significantly influences their mechanical properties and structural integrity. Some studies reported that drying the raw particles as a pretreatment improves board properties by optimizing moisture levels during hot pressing. Moisture acts as a plasticizer, lowering the glass transition temperature of biopolymers such as lignin, which softens and facilitates self-bonding at lower temperatures [62,73,114]. However, excessive initial moisture content can detrimentally affect board quality due to rapid vapor formation during hot pressing, leading to micro-fissures and blistering inside the boards. Hidalgo-Cordero et al. (2020) identified an optimal initial moisture content of approximately 2% across multiple lignocellulosic feedstocks to balance lignin plasticization and minimize defects [114]. Greater moisture content, particularly in materials containing pliable pith particles, resulted in increased blister formation due to restricted vapor escape and elevated internal pressure, impairing mechanical properties.

Almusawi et al. (2016) studied hemp shive particles and found that larger particles required higher moisture content (around 5%) to enhance flexural strength [60]. The moisture served as a plasticizer by softening lignin within particles and enabling its migration to the surface under applied load, while smaller particles showed less sensitivity to moisture variations. Moisture contents exceeding this level caused swelling and thermal degradation during pressing in closed molds due to high vapor pressure. These findings highlight the importance of precise moisture control, typically below 5%, to prevent localized overheating and preserve the performance of binderless boards during manufacturing.

In some research, water must be added to achieve a desired moisture content to generate steam during the thermocompression process, which contributes to extracting and plasticizing the lignocellulosic compounds contained [36,42,102].

In summary, the relation between temperature, pressure, pressing time, and moisture content governs the physicochemical transformations that enable self-bonding in lignocellulosic materials. Achieving optimal conditions is essential to activate lignin, control hemicellulose degradation, and maintain structural integrity. Precise regulation of these parameters minimizes defects, ultimately determining the mechanical performance and durability of binderless boards.

## 6. Selected Lignocellulosic Raw Materials for Binderless Board Production

The selection of raw materials for producing binderless boards plays a fundamental role in determining the mechanical and physical properties of the final product. Essential to self-bonding mechanisms are the intrinsic components of lignocellulosic materials (principally lignin, hemicelluloses, and cellulose) that undergo chemical and physical transformations during processing. Lignin softening and thermoplasticization facilitate the flow and polycondensation reactions that create natural adhesion between particles without added adhesives. Meanwhile, hemicelluloses and cellulose contribute structurally, with their modifications enhancing bonding strength and board integrity. Additionally, the presence of extractives and storage polymers such as starch and proteins can impact matrix cohesion and board performance by influencing chemical interactions during thermal treatment.

**Figure 6:**
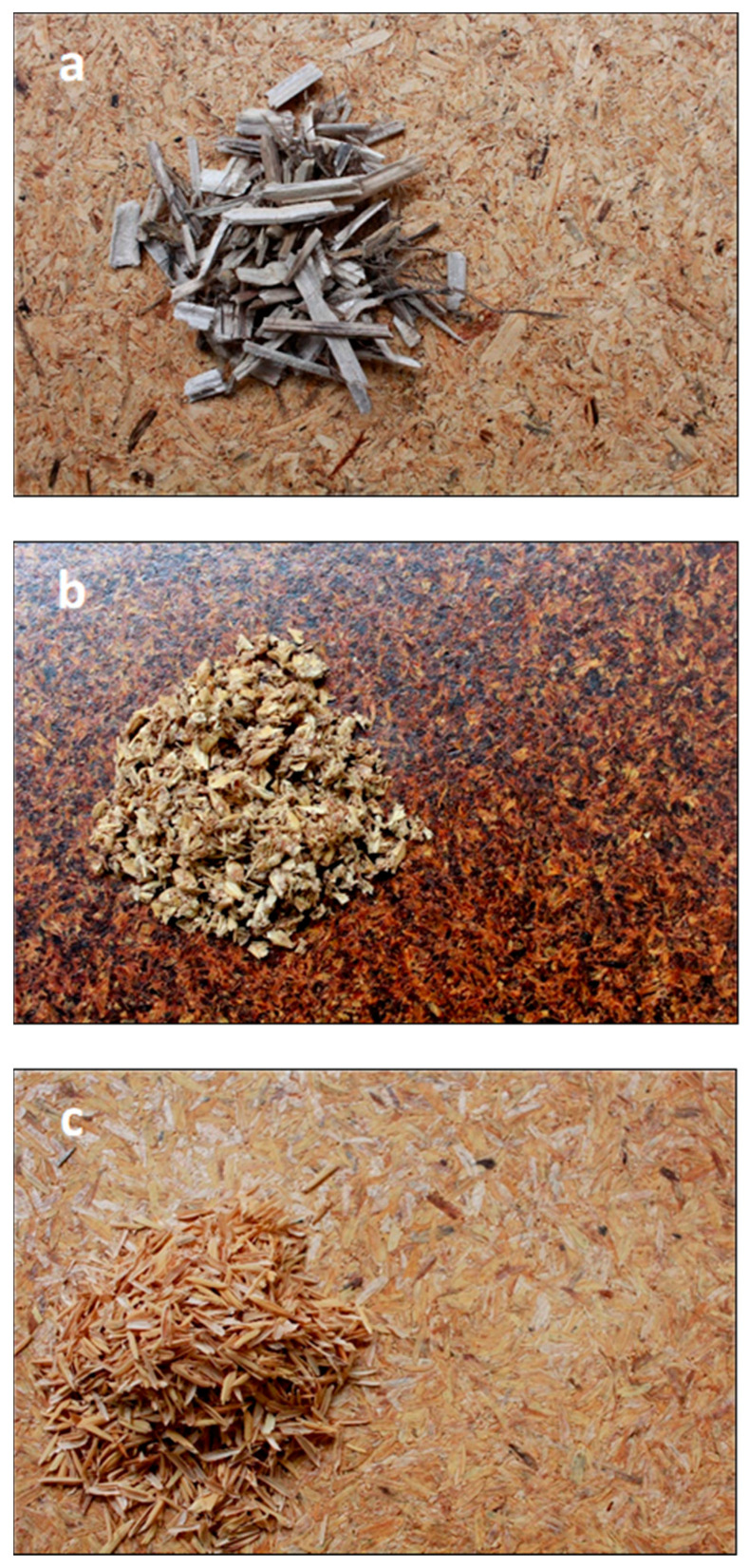
Boards from diverse agro-industrial residues: (**a**) hemp, (**b**) BSG, and (**c**) rice husk (own photo).

According to network analysis clusters “A” and “B”, common raw materials utilized in binderless board production include wheat straw, rice straw, kenaf, oil palm residues, coconut husk, sugarcane bagasse, bamboo, and cotton stalks, reflecting both economic viability and sustainability considerations [44,51,55,59,98,115,116]. On the other hand, Cluster “F” focuses on the diverse applications and valorization pathways of BSG, the primary by-product of the brewing industry. Figure 6, shows different boards from diverse agro-industrial residues [41,117,118]. The choice of material affects various quality attributes, including mechanical strength indicators such as MOR, MOE, and IB. Furthermore, particle size and board thickness influence water resistance, dimensional stability, and thermal and acoustic properties [29,31,32,73]. Optimizing these factors with suitable lignocellulosic feedstocks ensures enhanced performance of binderless boards aligned with environmental and cost-efficiency goals.

This section provides a brief overview of key features and properties of some raw materials that have been most extensively researched for binderless board production. Special emphasis will be placed on BSG as a raw material, with a discussion of its properties in binderless boards, as well as its applications, costs, and future perspectives in the following sections.

### 6.1. Rice Straw and Husk

Rice straw (RS) and rice husk (RH) are abundant agricultural residues that have been extensively studied as raw materials for binderless board production. RH, in particular, contains a high content of silica (15%) [92], which can enhance dimensional stability and water resistance but may reduce mechanical strength if not properly processed. RS, on the other hand, is more flexible and easier to process, making it suitable for boards requiring higher IB strength and surface smoothness [119].

For densities of 800 kg/m^3^ (Table 3) in RS, it was observed that thermal pretreatment improved the MOE, while similar values were obtained for the IB of the boards, with a lower MOR [61,77]. This decrease in MOR may be because its value is influenced not only by internal bonding and fiber morphology but also by particle size. Considering this, smaller particle sizes tend to promote higher MOR values (see Table 3), previously described in Section 4.1. In addition, Kurokochi et al. (2022) studied the effect of particle size during thermal pretreatment for the production of RS binderless boards, and the highest IB, MOR, and MOE were obtained for boards made from heat-treated chips (≤50 mm), compared to those that were treated with powder (≤1 mm), indicating that heat treatment is more homogeneous for chips than powder and that the effectiveness of the bond is based on the size of the particles to which the thermal treatment is applied [61].

Ferrandez-Garcia (2017) reported that reducing RS particle size (<0.25 mm) led to an improvement in the MOE, MOR, and IB of the particleboards [35]. Furthermore, it was shown that thickness swelling only depended on particle size. Wang et al. (2022) found that the strength of the boards could be due to use particles sizes <0.038 mm, with smaller shortest/longest aspect ratios of RH showing the greatest flexural strength value [33]. In addition, cohesiveness between particles of that size could be strong and promote packing a high density of particles in the molding process.

As previously described, it can be observed that treatment pretreatment (steam explosion) of RH or RS primarily improves the MOR of the boards [33,34].

### 6.2. Sugarcane Bagasse

As a by-product of sugarcane processing, sugarcane bagasse is a promising raw material for binderless board production (Table 1), due to its richness in sugar-containing compounds that facilitate natural self-bonding during hot pressing. The abundance of bagasse residues, especially in agricultural sectors like Brazil, India, and China, makes it a sustainable resource for particleboard manufacturing [120]. Although residual sugars in bagasse can interfere with synthetic resin bonding, they can caramelize during hot pressing to improve self-adhesion without added binders [31,38,69,77]. Binderless boards made from bagasse show mechanical properties compatible with commercial resin-bonded particleboards, yet they typically suffer from higher water absorption and thickness swelling due to the hygroscopic nature of the material [77].

The properties of binderless boards prepared from sugarcane bagasse are shown in Table 4. In all cases, the mechanical properties improved with increasing density, as expected [38,39].

When comparing the boards with a density of 800 kg/m^3^ [39,77], it was observed that higher processing temperatures result in boards with a greater MOE and IB, along with a slight decrease in the MOR value. Additionally, an increase in temperature up to 280 °C did not produce any observable change in MOR values.

Nonaka et al. (2013) studied the impact of pressing temperatures above 200 °C and found that they significantly influenced the properties of bagasse binderless boards, with improvements observed from 200 °C to 260 °C [39]. However, a significant decrease in IB values (by a factor of two, 0.5 MPa) was observed at 280 °C, presumably due to the thermal degradation in the core layer of the board and a prominent density depression. According to these results, they identified 260 °C as the optimum processing temperature.

Widyorini et al. (2005) and Nonaka et al. (2013) agreed that the chemical composition and the morphology of the particles of raw matter played a significant role in influencing the IB [38,39]. In fact, Nonaka et al. observed that chemical composition is the dominant parameter in IB values [39]. Widyorini et al. (2005) studied the effect of particle form and composition in bagasse pith and rind [38]. Table 4 shows that the pith has higher MOR values than the rind boards for hot pressing and steam injection processing [39]. They concluded that the pith particles were more easily deformed and packed more closely than the rind particles, thereby increasing the self-bonding area. A similar behavior had previously been described by Mobarak et al. (1982) [69]. Ahmad et al. (2019) also reported that the particle packing arrangement resulted in fewer void spaces and improved fiber bonding [95]. Widyorini et al. (2005) also observed that boards that were produced using the steam injection method required shorter pressing times than hot pressing to obtain similar mechanical properties, even with lower densities [38].

### 6.3. Coconut Coir and Husk

Previous studies have addressed different aspects related to the use of coconut natural fibers for binderless fiberboard manufacturing (Table 5) [46,47,102]. Stelte et al. (2023) highlighted the strength and versatility of coir from coconut as raw material in particleboard production, as well as the need to seek sustainable alternatives to the synthetic adhesives commonly used in the industry [49]. In another investigation, Van Dam et al. (2004) focused on the importance of the irreversible modification of lignin for the development of adhesive-free board manufacturing techniques [48]. They argued that the high temperatures used in fiber pretreatment may negatively affect the chemical reactivity of lignin, limiting the fibers’ ability to adhere to each other naturally. In the same line, Araújo et al. (2018) highlighted how high temperatures could lead to the degradation of cellulose and lignin, thereby affecting the strength and elasticity of the boards [46]. At temperatures over 220 °C, a high level of degradation was observed, resulting in damages and fragility on the surface of the coconut boards, reducing mechanical values of the fiberboards. For high-density boards from white coir fiber and pith, optimal pressing conditions were at 15.7 MPa at 220 °C, for 4 min, promoting the curing of the lignin in the materials and favoring the bonding between fibers.

### 6.4. Wheat Straw

Wheat straw is an abundant and low-cost lignocellulosic agricultural residue (Table 1) that is readily available after grain harvesting. Its high cellulose content and natural lignin make it a promising raw material for binderless boards, as these constituents contribute to the mechanical strength and self-adhesive bonding during hot pressing. Using wheat straw for binderless boards also contributes to environmental sustainability by valorizing agricultural waste that is otherwise often burned or discarded, reducing air pollution, and supporting circular economy principles. Additionally, wheat straw offers biodegradability, lightweight, and good thermal and acoustic insulation properties, making it an eco-friendly alternative to wood-based panels.

Moreover, wheat straw can be processed with relatively low energy input through steam explosion pretreatment, which partially degrades hemicelluloses and softens lignin, enhancing the self-bonding capability and dimensional stability of the resulting binderless boards. 

When evaluating thermal pretreatment, Tupciauskas et al. (2022) investigated the effect of steam explosion on wheat straw and observed a significant improvement in water uptake [20]. The material undergoes steam explosion pretreatment (e.g., at 220 °C for 2 min), which causes partial degradation of hemicelluloses and softening of lignin, allowing self-adhesive bonding during hot pressing. Binderless boards from steam-exploded wheat straw achieve mechanical properties with average values of 4482 N/mm^2^ for the MOE, 22.1 N/mm^2^ for the MOR, and 0.54 N/mm^2^ for the IB, as well as TS 9% and WA 35%, under optimized pressing conditions [59]. These values meet or exceed the minimum requirements of the EN 312 P3 standard for particleboards in humid conditions. Higher density boards (1000 kg/m^3^) show further improvements in mechanical strength, with MORs up to nearly 20 MPa (Table 6) and enhanced water resistance, confirming wheat straw’s viability for structural panel applications without synthetic adhesives. This beneficial impact is further supported by the literature, obtaining a much higher TS value when no pretreatment other than shredding was applied, compared to an average TS value of 9% in the steam-exploded samples [59,76]. Pintiaux et al. (2015) also noted that the IB improved under severe steam pretreatment conditions, attributing this enhancement to the generation of finer particles [73]. Additionally, Luo et al. (2012) demonstrated that steam explosion pretreatment effectively solubilized hemicellulose fractions, contributing to the modification of the biomass structure and potentially improving board properties [121]. 

Halvarsson et al. (2009) observed that for a wheat straw board density of approximately 1000 kg/m^3^, the Fenton pretreatment improved the MOE and IB properties, while the MOR decreased [36]. It is noteworthy how this chemical pretreatment improves the interactions between particles in the boards prepared without adhesives, as was described in previous sections. Oxidation pretreatment facilitated adhesive bonding between fibers through the activation of the fiber surface and intra-fiber crosslinks, thanks to the diffusion of low-molecular-weight components into the lumen of cell walls [29,36].

The study of binderless boards based on wheat straw further indicates that pressing temperature and time critically influence board performance; increasing these parameters improves inter-fiber bonding by promoting lignin condensation and crosslinking reactions. However, too-high temperatures or inadequate pressing time may cause defects such as blisters or cracks, especially at higher densities (1200 kg/m^3^) [20]. Chemical analysis by FTIR demonstrates significant hemicellulose deacetylation and lignin modification during pretreatment and pressing, which are crucial for self-bonding. Overall, wheat straw binderless boards represent a promising eco-friendly alternative that valorizes agricultural waste and eliminates formaldehyde emissions, though attention to pressing parameters is essential to balance strength and dimensional stability [20,59].

### 6.5. Oil Palm Trunk

Palm trunk, including oil palm (*Elaeis guineensis*), is considered a renewable and sustainable natural resource widely used as a cellulosic raw material in various panel products such as particleboard, medium-density fiberboard (MDF), cement-bonded particleboard, blockboard, plywood, and, notably, binderless boards. This broad utility is attributed to palm trunk’s abundant availability as an agro-industrial residue and its lignocellulosic composition, which supports self-bonding mechanisms essential for binderless board manufacture [18,84,90]. Different parts of the palm plant, including the core, mid-parts, fronds, bark, and leaves, have been explored for panel production. However, dimensional stability remains a key challenge due to the hygroscopic nature of non-wood fibers.

Research studies on binderless fiberboards from oil palm wastes demonstrate promising improvements in mechanical and physical properties by applying pretreatments such as steam explosion and steam pretreatment; see Table 7 [18,31]. In Mejía et al. (2014), binderless boards made from steam-exploded oil palm empty fruit bunches achieved favorable mechanical properties (MOR up to ~28 MPa and MOE ~3100 MPa) and dimensional stability that met Colombian standards, outperforming fiberboards treated with oxidative Fenton reagents [84]. In addition, the best results for fiberboards treated with the highest design conditions (pressing temperature of 190 °C) gave optimum values of WA 22.7% and TS 11.8%. Similarly, Boon et al. (2019) showed that steam pretreatment of oil palm trunk particles improved binderless particleboard properties by reducing hemicellulose and starch content while increasing cellulose concentration [90]. Longer steam pretreatment duration enhanced mechanical strength and dimensional stability, though performance still fell short of conventional particleboards bonded with synthetic urea–formaldehyde resins.

These studies underscore the potential of oil palm trunk and its residues as sustainable raw materials for binderless boards, leveraging their intrinsic lignin and cellulose for natural self-bonding. The steam-based pretreatments modify the chemical composition and fiber structure to promote stronger inter-particle bonding and water resistance. However, challenges such as achieving dimensional stability comparable to synthetic resin-bonded boards remain, motivating further research into optimizing pretreatment parameters and hybridizing with natural reinforcements for enhanced performance. Overall, palm trunk biomass offers a promising eco-friendly alternative to reduce formaldehyde emissions and valorize agricultural wastes in the panel industry.

### 6.6. BSG

The applications and valorization pathways of brewer’s spent grain, the primary by-product of the brewing industry, emerges prominently in cluster “F” of our citation network analysis [41,89,117,122]. BSG is a lignocellulosic by-product mainly composed of barley malt husks that is still used as low-value animal feed, reflecting underdeveloped value chains and low supplier capability compared to other bio-residuals. However, recent studies demonstrated that BSG can be successfully incorporated into high-value products, such as particleboards, bio-based panels, and low-carbon aggregates for construction applications. For example, Barbu et al. (2021) and Klímek et al. (2017) showed that partially replacing wood particles with BSG in adhesive-bonded particleboards yields viable non-structural panels [25,117]. El Haddaji et al. (2024) demonstrated the potential of BSG as a low-carbon aggregate in bio-based construction composites, while Ferraz et al. (2013) found that its inclusion in clay bricks reduces density and improves thermal insulation [123,124].

Moreover, BSG has emerged as a sustainable raw material for the production of binderless particleboards without the need for synthetic adhesives. Rossi et al. (2024) produced binderless boards entirely from BSG with acceptable mechanical and dimensional stability for use in interior cladding panels or ceilings applications [41]. BSG contains substantial amounts of hemicellulose, cellulose, lignin, and proteins (Table 1), which under specific hot-pressing conditions (particularly at 170 °C) and controlled particle sizes (0.2–2.38 mm) undergo chemical transformations such as hydrolysis, dehydration, and oxidation. As was described before, the processing temperature and particle size significantly influenced the board properties. Processing temperatures of 160 °C and 170 °C were tested; however, 180 °C was found unsuitable because it caused material loss and collapse due to rapid vapor generation that created voids and reduced adhesion. Boards pressed at 170 °C exhibited better particle-to-particle self-adhesion, leading to improved mechanical performance, including a higher MOR, MOE, and IB, compared to those processed at 160 °C (Table 8) [41].

Regarding particle size, smaller BSG particles (0.2–2.38 mm), achieved by grinding, resulted in broader particle size distribution and greater particle surface area, which enhanced mechanical interlocking and chemical bonding during hot pressing. This was evidenced by significantly higher MOR, MOE, and IB values in boards made with ground BSG at 170 °C compared to larger raw particles. The improved particle contact increased the effective bonding area, yielding boards with superior structural integrity and water resistance. FESEM images confirmed more compact particle packing and fewer voids in these boards, demonstrating the crucial role of particle size in optimizing the self-adhesion process for binderless BSG particleboards [41].

Compared to conventional wood-based boards or those using synthetic phenolic resins, BSG binderless boards demonstrate competitive properties suitable primarily for non-structural interior applications, offering an eco-friendly alternative by eliminating toxic formaldehyde emissions associated with synthetic adhesives.

Due to its high composition (Table 1) and intrinsic porosity, BSG exhibits moderate thermal insulation capability. Mussatto et al. (2006) and Lynch et al. (2016) summarized its composition, noting high hemicellulose and lignin contents that contribute to low thermal conductivity (λ) compared with synthetic fillers [125,126]. When compared to other agro-industrial residues (see Table 9), hemp and flax show lower λ values (~0.05–0.08 W/m·K), while BSG-based panels tend to exhibit λ ≈ 0.10–0.13 W/m·K [127,128]. Such performance is adequate for internal thermal insulation, though still higher than mineral wool or polystyrene foams (typically 0.03–0.045 W/m·K) [129,130]. Recent studies suggest combining BSG with lighter bio-aggregates to improve its insulation efficiency [123,128].

Although BSG’s relatively low cellulose content limits stiffness compared to wood or other lignocellulosic residues, the use of BSG alone as both the fiber source and adhesive fulfills circular economy principles by valorizing agro-industrial waste streams. Ongoing research suggests potential improvements by reinforcing BSG cores with natural fibers or fabrics to develop mechanically stronger panels, broadening the material’s applicability toward structural uses [41,117]. This aligns with the increasing trend in exploring binderless boards from diverse agricultural residues for environmentally sustainable construction materials.

## 7. Costs Analysis and Future Perspectives: BSG Case

Brewer’s spent grain was selected as a representative case study for the cost analysis. As stated before, BSG appears in cluster F in our citation network analysis and represents the largest solid by-product of the brewing industry, generating around 39 million tons annually worldwide (~20 kg per 100 L beer), of which more than 6.4 Mt per year are generated in Europe alone [131]. However, special costs arise mainly from BSG’s high moisture content, logistics of collection and drying, and processing requirements that differ from conventional wood-based materials.

**Figure 7:**
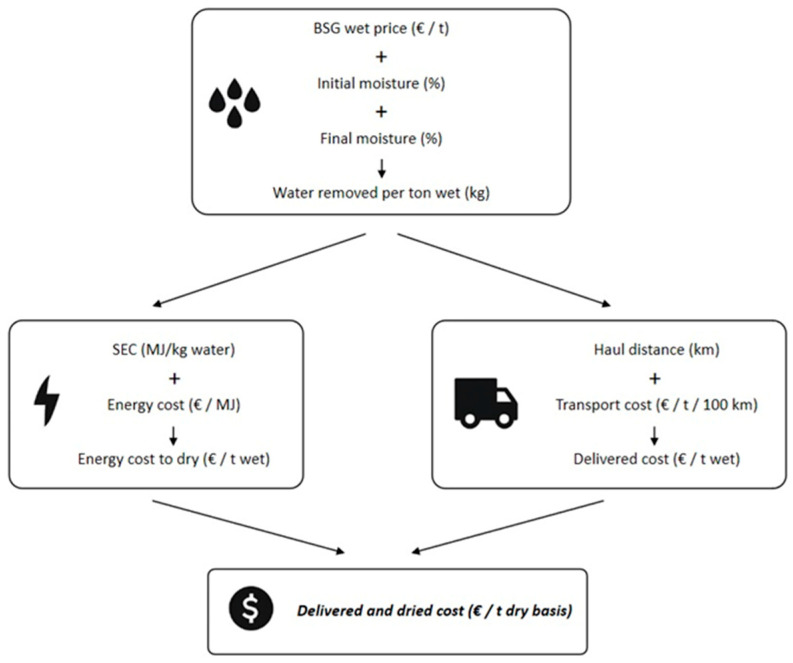
Cost calculation scheme.

Reported gate values for wet BSG are low, often around EUR 35–40 per ton and sometimes even zero (0 EUR t^−1^ wet basis). Its high moisture content (typically 70–85%) leads to rapid microbial spoilage and increased logistic costs. Transporting wet BSG is therefore uneconomical beyond ~200 miles due to this high-water content. The drying cost is mainly governed by the specific energy consumption (SEC), i.e., the energy required to evaporate 1 kg of water. To determine a delivered-and-dried cost (EUR/t, on dry basis) for BSG used in binderless panel production, a combination of moisture data, drying energy requirements (SEC + local energy tariff), and logistics parameters are required. The details of the raw cost estimations are provided in Section S3 of the Supplementary Information File and summarized in Figure 7. Binderless panels are usually processed by thermo-compression at ~180–200 °C. Steam-assisted systems may use 1–3.5 MPa to enhance bonding. Press energy rises with cycle time and pressure, with some reports exceeding 39 kWh/m^2^ at 200 °C. When employing steam injection or steam explosion pretreatments (1–3.5 MPa, ~190 °C), the steam generation and equipment energy burden should also be included [132]. The resulting costs can be then benchmarked against typical resin-bonded particleboard systems, where adhesives represent 30–50% of material cost (and up to ~32% of total manufacturing costs in glued-wood composites) [14]. A study of a European producer reported raw materials ≈ 2/3 the cost of the product sold, energy ~10%, personnel ~11%, and depreciation ~5%. This is for conventional particleboard; binderless will shift shares by removing resin but raising utilities [133] (UF resin pricing in 2025 reports is in the hundreds of USD/t, region-dependent). As shown in the schematic cost calculation pipeline of Figure 7, the final trade-off heavily depends on SEC, local tariffs, and supply chain logistics.

By using the previous calculation workflow and typical parameters, for 1 ton of BSG at 75% initial moisture and 10% final moisture, Δ_Mw_ ≈ 722 kg. Using SEC = 4–8 MJ kg^−1^ H_2_O, the drying cost is approximately EUR 60–115 t^−1^ wet. Adding EUR 1 t^−1^ transport (20 km at a rate of EUR 5/100 km) and assuming free BSG, the delivered-and-dried cost lies approximately within the EUR 250–450 t^−1^ range (dry basis, scenarios 1 and 2 in Table 10). Assuming a scenario in which BSG is processed immediately after collection (scenario 3 in Table 10), the final moisture content would be around 35%. This moisture level is sufficient because water is added during the thermocompression process to generate steam, which contributes to extracting and plasticizing the lignocellulosic compounds contained in the BSG. Partial drying to this level would reduce the drying cost, resulting in an estimated delivered-and-dried cost of approximately EUR 200–400 t^−1^ on a dry basis. The sensitivity analysis in Table 10 is based on the previous calculations and highlights the dominant influence of the drying stage on the overall cost of BSG valorization. Variations in dryer efficiency and energy tariffs lead to differences of more than twofold in the final dry-basis cost (see other complementary scenarios in Table S3, Section S4 of the Supplementary Information File). In contrast, logistical factors such as transport distance and gate price, though relevant at industrial scale, exert comparatively smaller effects. These results underline that any technological improvements in dewatering, heat recovery, and low-temperature drying have the greatest potential to enhance the economic viability of binderless board production from BSG. All estimated costs exclude capital expenditure, labor, and maintenance, which depend strongly on plant scale. The resulting range demonstrates that drying energy is the key determinant of BSG valorization economics and highlights the need for integrated heat management strategies in binderless board production.

### Future Perspectives for BSG Valorization

The literature shows that while progress has been achieved in understanding BSG composition, processing, and end-use potential, significant gaps persist between laboratory research and commercial implementation. In line with the observed cost structure, the most promising research directions involve the development of energy-efficient drying and dewatering to reduce production costs [134], exploration of hybrid pretreatments combining mechanical disintegration with enzymatic hydrolysis for improved fiber activation, design of bio-based compatibilizers or coupling agents to enhance particle bonding and moisture resistance, and techno-economic assessments integrating carbon accounting and life-cycle analysis to benchmark BSG panels against conventional boards.

Recent work demonstrates that BSG-based binderless panels can attain acceptable water resistance when carefully optimized for pressing temperature and particle size. Rossi et al. (2024) reported that pressing BSG at 170 °C with particle sizes between 200 and 2380 µm improved water resistance through enhanced particle–particle adhesion [41]. However, Barbu et al. (2021) and Klímek et al. (2017) observed that partial substitution of wood with BSG in adhesive-bonded boards may reduce mechanical strength and dimensional stability, likely due to differences in surface chemistry and particle morphology [25,117]. Future studies should therefore target pre-conditioning methods (e.g., fiber size control, hot-water or mild alkaline washing) to increase surface compatibility and reduce swelling sensitivity under humid conditions.

Pretreatment remains the most active research front in BSG valorization. Zeko-Pivač et al. (2022) emphasized that sustainable fractionation is essential to access fermentable hydrolysates and value-added chemicals, highlighting options such as acid hydrolysis, alkaline oxidation, steam explosion, and enzymatic hydrolysis [118]. Chetrariu (2020) detailed solvent-based and advanced extraction technologies including microwave-assisted, ultrasound-assisted, and supercritical CO_2_ extraction, each offering selective recovery of proteins, phenolics, and fibers [128].

Mechanical reinforcement and added functionalities depend strongly on BSG content and processing route. Barbu et al. (2021) showed that a 10% BSG replacement in wood particleboards may reach P2-grade classification (EN-312) for non-structural interior use, whereas higher loadings (>30%) lead to decreased bending strength and internal bond strength due to insufficient interfacial adhesion [117]. Conversely, the high protein and fiber content of BSG supports diverse biotechnological applications. Puligundla et al. (2021) and Zeko-Pivač et al. (2022) noted that solid-state fermentation can increase amino acids (≈2×), unsaturated fatty acids (≈1.7×), and antioxidant capacity (≈5×) compared with raw BSG [118,135]. Such improvements could be exploited for multifunctional bio-composites combining structural and antimicrobial or antioxidant performance.

Industrial scale-up continues to be the main constraint. Comprehensive cost–energy analyses and performance benchmarking remain scarce, impeding the consolidation of BSG as a consistent industrial feedstock. Zeko-Pivač et al. (2022) pointed out that although the European brewing sector could generate over 8.5 Mt yr^−1^ of BSG by 2030, economically viable valorization requires optimization of logistics, drying, and process integration [118]. Strategic partnerships between breweries and bio-based product manufacturers, coupled with policy incentives for waste valorization, will be crucial to move beyond pilot-scale demonstrations.

## 8. Conclusions

The shift toward sustainable panel technologies has driven extensive research on binderless and self-bonded lignocellulosic composites, which rely on natural bonding mechanisms rather than synthetic adhesives. In line with circular economy principles that promote recyclability and reduced raw material consumption, binderless fiberboards from unconventional residues represent a promising alternative to traditional wood panels. Using a complex network approach, this review mapped the scientific landscape of binderless board production, highlighting key research themes, such as processing parameters and pretreatment strategies. The cost analysis, valorization challenges, and future research directions identified for brewer’s spent grain can be extended to other lignocellulosic residues and undervalued agro-industrial by-products, including cereal husks and straws, sugarcane bagasse, oil palm trunk, and coconut coir. These feedstocks share similar compositional complexity and processing constraints, yet their abundance and renewable nature offer substantial potential for binderless boards, biopolymers, and multifunctional composites, as long as processing is optimized and integrated within circular bioeconomy frameworks.

## Figures and Tables

**Table 2:** Effect of steam explosion pretreatment on binderless board properties.

Raw Material	Pretreatment Method	Temperature (°C)/Time (min)	MOR (MPa)	IB (MPa)	WA (%)/TS (%)
*Arundo donax* L. [101]	Steam explosion	210/9.5	40.4	1.3	17.6/15
	control	-	40	0.7	75/65
Oil palm trunk [90]	Steam treatment	200/60	18.7	0.75	nd/21.8 (TS)
	control	-	15.4	0.66	nd/26 (TS)
Rice straw [103]	Steam treatment	200/20	6	0.32	35/5
	control	-	3	0.03	53/35

nd: no data.

**Table 3:** RS and RH binderless board processing parameters and properties obtained from the literature.

Raw Material	Pretreatment	TP (°C)	tP (min)	PP (MPa)	Particle Size (mm)	Density (kg/m^3^)	MOR. (MPa)	MOE (GPa)	IB (MPa)
RS [77]	-	180	10	n.d.	0.84	800 (^1^)	13	1.03	0.3
RS [61]	Heat treatment (200 °C, 1.5 MPa 40 min)	220	10	5	≤1	800 (^1^)	7.3	2.10	0.3
RH [33]	Hot compressed water (160 °C)	170–245	8	n.d.	n.d.	1400	21	2.37	n.d.
RS [35]	-	110	60 (3 cycles)	2.6	<0.25 mm	1101.5	18.02	2.58	0.3
RS [34]	Thermo-mechanical fractionation, twin-screw extruder; (100–110 °C) Biolignin™	200	5–1 (2 cycles)	22.3 (^2^)	-	1414	50.3	8.06	n.d.

(^1^) Target density reported, not measured; (^2^) pressure expressed as the force per unit area of molded fiberboard. n.d.: no data.

**Table 4:** Sugarcane bagasse binderless board processing parameters and properties obtained from the literature. BP: Bagasse pith. BR: Bagasse rind.

Raw Material	Pretreatment	TP (°C)	tP (min)	PP (MPa)	Density (kg/m^3^)	MOR (MPa)	MOE (GPa)	IB (MPa)
BP and residual sugar [77]	-	180	10	n.d.	800	20	1.9	0.6
BP, BR [39]	-	260	10	n.d.	800	16.6	3.5	1.2
Depithed bagasse [69]	<0.5 mm *	175	5	25.5	1350	63	-	-
BP [38]	-	190	10	n.d.	750	10	1.4	0.2
BR [38]	-	190	10	n.d.	750	2.0	0.4	0.1
BP [38]	Steam injection	190	3	1.0	650	6.0	0.8	0.15
BR [38]	Steam injection	190	3	1.0	650	2.0	0.4	0.09

* Particle size.

**Table 5:** Coconut-based binderless board pretreatments, processing parameters, and properties obtained from the literature.

Raw Material	Particle Size (mm)	TP (ᵒC)	tP (min)	PP (MPa)	Density (kg/m^3^)	MOR (MPa)	MOE (MPa)	IB (MPa)
Coconut coir [48,102]	0.88	180	3–30	0.30–0.75	1300–1400	50	5000	nd.
Coconut husk [78]	8–10	200	13	14.7	450	1.94	365	0.002
White coir pith and fiber 7:3 (*w*/*w*) [46]	4	210	4	15.7	1372	18	3410	nd.
Coconut coir [47]	10–20	180	20	2.4	500	37	2800	0.31

nd: no data.

**Table 6:** Wheat straw-based binderless board pretreatments, processing parameters, and properties obtained from the literature.

Pretreatment	TP (°C)	tP (min)	PP (MPa)	Density (kg/m^3^)	MOR (MPa)	MOE (MPa)	IB (MPa)
Fenton [36]	200	1.5	0.5	1000	28	4000	0.5
Soda pulping (7 wt% NaOH) [37]	150/220	45/10	8/14	1013	52.8	1890	0.4
Mechanical refining [37]	150/220	45/10	8/14	1119	98.7	6500	1.6
Steam explosion [59]	175	16	6	1150	22.1	4482	0.6
Steam explosion [20]	175	16	6	800	15.5	2750	0.6

**Table 7:** Pretreatments, processing parameters, and properties obtained from the literature for oil palm trunk boards without adhesives.

Pretreatment	TP (°C)	tP (min)	PP (MPa)	Density (kg/m^3^)	MOR (MPa)	MOE (MPa)	IB (MPa)
Steam pretreatment (160 °C) [90]	200	20	10	600	18.7	n.d.	0.75
Hot water (100 °C, 30 min) [18]	200	25	15	650	8.18	n.d.	n.d.
Steam explosion and Fenton reagent oxidation [84]	190	6	5	1200	28.5	3100.1	n.d.

nd: no data.

**Table 8:** Pretreatments, processing parameters, and properties obtained from the literature for BSG boards without adhesives. PP: 2.1 MPa; tP: 15 min [41].

Particle Size (mm)	TP (°C)	MOR (MPa)	MOE (GPa)	IB (MPa)
>2.5	160	1.82	0.46	0.09
>2.5	170	2.43	0.54	0.15
0.2–2.38	160	2.06	0.61	0.09
0.2–2.38	170	4.14	0.77	0.23

**Table 9:** Comparison of insulation materials.

Material	Thermal Conductivity (W/m·K)	Density (kg/m^3^)	Typical Applications
BSG panel	0.10–0.13	350–450	Interior insulation, furniture cores
BSG–hemp mix	0.07–0.10	250–350	Partition boards, eco-panels
Hemp shive panel	0.05–0.08	220–300	Light insulation, acoustic boards
Mineral wool	0.035–0.045	30–100	Thermal and acoustic insulation
Expanded polystyrene (EPS)	0.031–0.038	15–30	Conventional wall/roof insulation

**Table 10:** Sensitivity to parameters in cost analysis.

Parameter	Scenario 1. *High-Efficiency or Heat-Recovery System (Most Energy-Efficient Drying Scenario). No Gate Prices, Low Haul Distance, and Cheap Energy Cost.*	Scenario 2. *Same as 1, but Conventional Convective Dryers with Limited Heat Recovery.*	Scenario 3. *Same as 1, but Just Drying Up to Processing Conditions (35%).*
BSG wet price (€/t)	0	0	0
Initial moisture (%)	75	75	75
Final moisture (%)	10	10	35
Water removed per ton wet (kg)	722.2	722.2	615.4
Specific energy consumption (SEC, MJ/kg water)	4	8	4
Energy cost (€/MJ)	0.02	0.02	0.02
Energy cost to dry (€/t wet)	57.8	115.6	49.2
Haul distance (km)	20	20	20
Transport cost (€/t/100 km)	5	5	5
Delivered cost (EUR/t wet, incl. transport)	1	1	1
Delivered and dried cost (EUR/t dry basis)	235.1	466.2	200.9

## Data Availability

The original contributions presented in this study are included in the article/Supplementary Materials. Further inquiries can be directed at the corresponding author.

## Supplementary Materials

The following supporting information can be downloaded at: https://www.mdpi.com/article/10.3390/polym17223082/s1, Figure S1: PageRank centrality illustration showing the random-walker probability used to identify influential papers in the network. Figure S2: Detected clusters (A–G) in the citation network obtained with the Louvain modularity optimization method (Blondel et al., 2008). Table S1: Seed articles used to construct the complex citation network. Table S2: European industrial costs for process heat and electricity (2023–2024) with conversion to €/MJ. Table S3: Sensitivity of delivered-and-dried cost to key parameters (scenarios 4–7).

## Abbreviations

The following abbreviations are used in this manuscript:

TP	Pressing Temperature
tP	Pressing time
PP	Pressing Pressure
MOE	Modulus of Elasticity
MOR	Modulus of Rupture
IB	Internal Bond strength
TS	Thickness Swelling
WA	Water Absorption
BSG	Brewer Spent Grain
VOCs	Volatile Organic Compounds
FAO	Food and Agriculture Organization
