# Supplementary material for: From Agricultural Residues to Sustainable Boards: Complex Network Analysis of Binderless Composites
Source: Polymers (Basel). 2025 Nov 20;17(22):3082. doi: 10.3390/polym17223082 (PMC12655928; doi:10.3390/polym17223082)
Supplement: Supplementary file 1 [file polymers-17-03082-s001.zip › polymers-3978211-supplementary.pdf]

SUPPLEMENTARY INFORMATION

FROM AGRICULTURAL RESIDUES TO SUSTAINABLE BOARDS: COMPLEX NETWORK ANALYSIS OF BINDERLESS COMPOSITES

L. Rossi[1], L.A. Miccio[1,2*], E.M. Ciannamea[1], P.M. Stefani[1]

[1] *Institute of Materials Science and Technology (INTEMA), National Research Council (CONICET), Colón 10850, 7600 Mar del Plata, Buenos Aires, Argentina.*

[2] *Departamento Polímeros y Materiales Avanzados: Física, Química y Tecnología, University of the Basque Country (UPV/EHU), P. Manuel Lardizábal 3, 20018 San Sebastián, Spain.*

SI 1. Knowledge map construction

We constructed a complex network of scientific publications by selecting seed articles (see Table S1) representative of binderless boards, bio-based adhesives, and agro-industrial residue utilization. Using their Digital Object Identifiers (DOIs), we extracted metadata and citation information from crossref.org.

Table S1. Seeds for complex network construction

Title	*Author*	*Journal, DOI, Year*
Lignin plasticization to improve binderless fiberboard mechanical properties	*Bouajila et. al.*	*Polymer Engineering & Science, 10.1002/pen.20342, 2005*
Chemical changes of kenaf core binderless boards during hot pressing (I): influence of the pressing temperature condition	*Okuda et. al.*	*Journal of wood science, 10.1007/s10086-005-0761-4, 2006*
Binderless fiberboard from steam exploded banana bunch	*Quintana et. al.*	*Industrial Crops and Products, 10.1016/j.indcrop.2008.04.007, 2009*
Application of agro-waste for sustainable construction materials	*Mangesh et. al.*	*Construction and Building Materials, 10.1016/j.conbuildmat.2012.09.011, 2013*
A review of preparation of binderless fiberboards and its self-bonding mechanism	*Zhang et. al.*	*Wood Science and Technology, 10.1007/s00226-015-0728-6, 2015*
Recent development in binderless fiber-board fabrication from agricultural residues	*Mahmood et. al.*	*Construction and Building Materials, 10.1016/j.conbuildmat.2019.03.279, 2019*
Brewer's Spent Grains: Possibilities of Valorization	*Chetrariu et. al.*	*Applied Sciences, 10.3390/app10165619, 2020*
Binderless fiberboards for sustainable construction. Materials, production methods and applications	*Vitrone et. al.*	*Journal of building engineering, 10.1016/j.jobe.2021.102625, 2021*
Alternative lignocellulosic raw materials in particleboard production	*Pędzik et. al.*	*Industrial Crops and Products, 10.1016/j.indcrop.2021.114162, 2021*
Particleboard from Agricultural Biomass and Recycled Wood Waste	*Seng Hua Lee et. al.*	*Journal of materials research and technology, 10.1016/j.jmrt.2022.08.166, 2022*
Development and performance of particleboard from various types of organic waste and adhesives	*Baharuddin et. al.*	*International Journal of Adhesion and Adhesives, 10.1016/j.ijadhadh.2023.103378, 2023*
Physical and Mechanical Properties of High-Density Fiberboard Bonded with Bio-Based Adhesives	*Gumowska et. al.*	*Forests, 10.3390/f14010084, 2023*

These networks were then merged into a unified network (duplicates were removed to avoid redundancy), resulting in a consolidated network with over 13000 nodes and nearly 20000 edges. PageRank was used to find influential works (as shown in Figure S1, it identifies those that are consistently cited across the network, signaling their long-term influence).

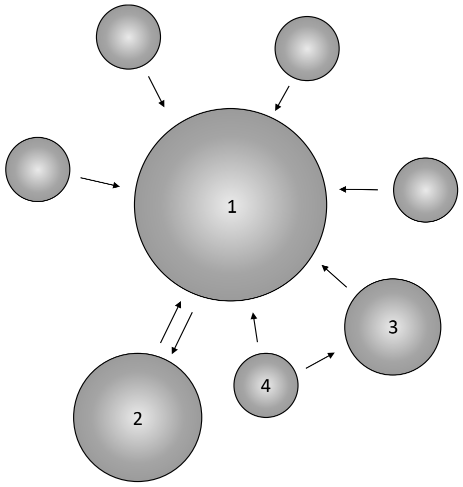

Figure S1. PageRank assigns a probability distribution representing the likelihood that a random walker would land on a particular node at any given time. This probability is calculated iteratively, taking into account both the number and the quality of links, with links from highly ranked nodes contributing more to the score. Thus, nodes with higher PageRank values are considered more central (for example node "1") or influential within the network.

In this way, the network provides a data-driven knowledge map, where nodes represent publications and directed edges represent citation links. The resulting map reveals several major clusters. The binderless boards and self-bonded lignocellulosic composites cluster emerges as a central hub, closely interconnected with clusters on bio-based adhesives, thermal treatments and alternative raw materials, pretreatment strategies, particleboards from agricultural residues, and applications of brewer's spent grain. This structure reflects the multidisciplinary nature of the field, where innovations in chemistry, process engineering, and biomass utilization converge to support the development of sustainable board technologies.

SI 2. Clusterization and content analysis.

Clustering analysis was performed in Gephi 0.9 using the modularity optimization algorithm, also known as the Louvain or fast-unfolding method (see Figure S2). This algorithm seeks to maximize the modularity score, which quantifies the density of links within clusters compared to links

between clusters. In our case, the algorithm yielded a modularity value of 0.816, confirming the presence of a strong community structure in the network. Each resulting modularity class corresponds to a cluster of closely related publications, labelled A to G in our knowledge map (see Figure 2 in main manuscript). These clusters represent emergent research fronts d efined directly by citation patterns rather than by manual grouping, ensuring an objective and reproducible mapping of the literature. The detected communities were then characterized using word frequency analysis of paper titles and abstracts, providing insight into the thematic focus of each cluster.

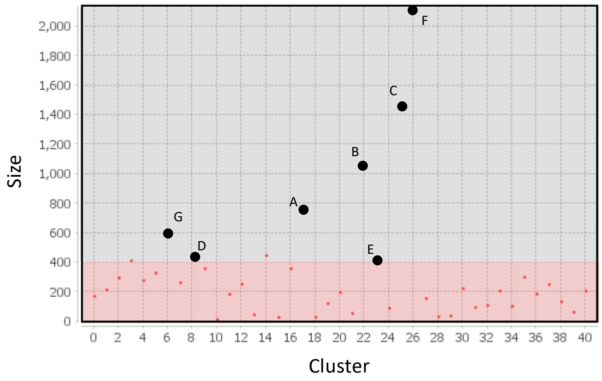

Figure S2. Cluster in the complex network, A to G correspond to the labels in the analyzed knowledge map below. Clusterization was performed by following ***VD Blondel et. al., "Fast unfolding of communities in large networks", in Journal of Statistical Mechanics: Theory and Experiment 2008 (10), P1000****.*

Word clouds were generated to visualize the dominant concepts and recurring terms within each thematic area. For example, in cluster "A" (see Figure 2 in main manuscript), words such as lignin, pretreatment, pressing temperature, and agro-residues emerged as central. This combination of network metrics and text mining allowed us to objectively characterize the research fronts and knowledge gaps across the field.

A) This cluster shows research on the production of binderless boards and particleboards derived from agricultural residues, wood by-products, and lignocellulosic biomass. The focus is on exploiting natural self-bonding mechanisms (particularly lignin softening, thermoplasticization, and enzymatic or steam-assisted pretreatments) rather than using synthetic resins. Materials explored include wheat straw, rice straw, kenaf, oil palm, coconut husk, bagasse, bamboo, cotton stalks, and other plant residues. Studies investigate processing parameters (pressing temperature, pressure, time, density), pretreatments (steam explosion, fungal or enzymatic modification, laccase oxidation, heat treatment), and the role of lignin chemistry in adhesion.

B) These papers mainly examine the use of diverse agricultural by-products, wood waste, and non-wood biomass as alternative raw materials for particleboard and fiberboard production. Materials investigated include coconut husks, bamboo, banana pseudo-stems, sugarcane bagasse, walnut and almond shells, date palm fronds, roselle stalks, tea leaves, tobacco stalks, and grapevine residues, among others. Studies highlight the influence of particle size, density, adhesives (such as castor oil polyurethane, starch, and modified formaldehyde resins), and additives (e.g., nanoparticles, vermiculite, metals) on mechanical strength, durability, acoustical performance, termite/fungal resistance, and emissions.

C) Papers in this group study the development and performance of adhesives derived from natural polymers and modified proteins for wood and particleboard applications. A strong emphasis is placed on soy protein systems, including chemical modifications (urea, guanidine, alkali, cross-linking) to improve bonding strength, hydrophobicity, and water resistance. Complementary studies address lignin-, tannin-, starch-, and chitosan-based adhesives, as well as blends and hybrid systems with urea–formaldehyde resins or pMDI. Research also explores the reduction of formaldehyde emissions, the incorporation of agro-residues such as rice husks, bagasse, and straw, and the role of chemical treatments like nitric acid activation. Together, these works highlight advances in eco-efficient, formaldehyde-free adhesive alternatives.

D) This collection focuses on the use of laccases and laccase–mediator systems to modify lignocellulosic fibers for sustainable wood composites and pulp applications. Topics include enzymatic activation of fibers for enhanced adhesion and self-bonding, wet-strength improvement of kraft pulp, and eco-friendly fiberboard production without synthetic binders. Several studies examine the reactivity of bacterial and fungal laccases with lignin, while others highlight their role in surface functionalization, grafting, and environmentally friendly bleaching processes.

E) This group of papers explore the role of thermal modification and alternative raw materials in enhancing wood composites. Research includes thermal rectification of eucalyptus, OSB pre-treatments, and oil-curing methods applied to particleboards from rubberwood, oil palm, sugarcane bagasse, and recycled pine. At the same time, novel feedstocks such as totora reeds, maize cobs, soybean residues, coconut waste, and bagasse are tested for panel production and functional uses like sound absorption. Hybrid composites combining residues with traditional fibers, for example malva or eucalyptus blends, further demonstrate how thermal processes and diverse biomass resources can improve durability, sustainability, and application versatility.

F) This cluster explores the diverse applications and valorization pathways of brewer's spent grain, the primary by-product of the brewing industry. Research spans from material science (bio-composites, films, foams) to food and feed applications (functional foods, bakery

enrichment, aquaculture, livestock nutrition), bioactive compound extraction (polyphenols, peptides, proteins), and biorefinery routes (ethanol, xylitol, lactic acid, carboxylic acids, biochar, volatile fatty acids).

G) This group covers a wide range of physical, chemical, physicochemical, and biological pretreatment methods applied to lignocellulosic biomass to improve enzymatic digestibility and conversion efficiency. The works examine steam explosion, alkaline peroxide delignification, NaOH wet-state treatment, hydrothermolysis, laccase oxidation, fungal degradation, and mechanical processing. Case studies include olive tree wood, corn stover, poplar, wheat straw, beech, sunflower stalks, sugarcane bagasse, and Jerusalem artichoke. Beyond pretreatment, the papers also discuss downstream outcomes such as sugar release, saccharification, biogas/biogasification, biohydrogen production, and the potential of dedicated energy crops.

SI 3. Raw cost estimations

To determine a delivered-and-dried cost (€/t, on dry basis) for BSG used in binderless panel production, a combination of moisture data, drying energy requirements, and logistics parameters is required: ***a) Moisture content:*** the initial BSG moisture content (X_i) could typically oscillate around 70-85%[132]. By selecting a target final moisture X_f ≈10% (suitable for storage) or X_f ≈35% (suitable for processing), the mass of water (Kg) removed per ton of wet BSG can be calculated as $\Delta_{Mw} = 1000 - [1000(1 - X_i) / (1 - X_f)]$; ***b) SEC:*** conventional convective dryers show 3.6-7.2 MJ kg^{-1} H_2O, while heat-pump dryers can reach ≈1.2 MJ kg^{-1} H_2O[133]. By multiplying Δ_{Mw} by the approximated SEC, the total drying energy (E_{dry}, MJ t^{-1} wet) can be estimated; ***c) Local energy tariff:*** multiply E_{dry} by the site-specific tariff (P_{energy}, €/MJ or €/kWh) to obtain the drying cost per ton wet: $C_{energy} = E_{dry} \times P_{energy}$ (as shown in Table S2 typical electricity/heat costs are approximately € 0.02-0.04 MJ^{-1});

Table S2. European industrial costs for process heat or electricity over 2023-2024.

Source	Data	Conversion to €/MJ	Notes
**Eurostat "Energy prices for non-household consumers" (dataset nrg_pc_205, 2023-2024)***	EU-27 industrial **electricity prices** ≈ € 0.08-0.14 kWh^{-1} (medium consumers, excluding VAT)	0.08 €/kWh ÷ 3.6 = **€ 0.022 MJ^{-1}** to 0.14 €/kWh ÷ 3.6 = **€ 0.039 MJ^{-1}**	Official European Commission statistics
**IEA Industrial Energy Prices (2023)**	**Natural gas for industry** ≈ € 0.02-0.03 kWh^{-1}	0.02 €/kWh ÷ 3.6 = **€ 0.0055 MJ^{-1}** to 0.03 €/kWh ÷ 3.6 = **€ 0.008 MJ^{-1}**	When thermal drying uses gas or steam rather than electricity

* https://ec.europa.eu/eurostat/databrowser/view/nrg_pc_205/default/table?lang=en

d) Logistics: transport cost can be estimated from the haul distance (d, km) and rate (r, €/t per 100 km) as $C_{transport} = r \times (d/100)$. BSG transport is only economical for short wet-haul radii (<200 miles)[134]. The delivered wet cost becomes $C_{wet,deliv} = P_{BSG,wet} + C_{transport}$, where $P_{BSG,wet}$ is the gate price (often € 0-40 t^{-1} wet); ***e) Dry-mass basis:*** the total cost per ton dry is obtained by dividing the sum of delivered and drying costs by the dry-mass fraction $C_{deliv\&dried} = (C_{wet,deliv} + C_{energy}) / (1 - X_i)$.

SI 4. Cost sensitivity.

Variations in dryer efficiency and energy tariffs lead to differences of more than twofold in the final dry-basis cost. In contrast, logistical factors such as transport distance and gate price, though relevant at industrial scale, exert comparatively smaller effects (see scenarios 4 to 7 in Table S3 below). These results underline that any technological improvements in dewatering, heat recovery, and low-temperature drying have the greatest potential to enhance the economic viability of binderless board production from BSG.

Table S3. Sensitivity to parameters in cost analysis

**Parameter**	**Scenario 4.** Same as 1, but breweries charge for removing their residue (positive gate price), the feedstock cost adds directly to the total.	**Scenario 5.** Same as 2, but more expensive energy.	**Scenario 6.** Same as 5, but longer distance.	**Scenario 7.** Same as 6, but larger water content (85%).
BSG wet price (€ / t)	40	0	0	0
Initial moisture (%)	75	75	75	85
Final moisture (%)	10	10	10	10
Water removed per tonne wet (kg)	722.2	722.2	722.2	833.3
Specific energy consumption (SEC, MJ/kg water)	4	8	8	8
Energy cost (€ / MJ)	0.02	0.04	0.04	0.04
Energy cost to dry (€ / t wet)	57.8	231.1	231.1	266.7
Haul distance (km)	20	20	200	200
Transport cost (€ / t / 100 km)	5	5	5	5
Delivered cost (€ / t wet, incl. transport)	41	1	10	10
**Delivered and dried cost (€ / t dry basis)**	**395.1**	**928.4**	**964.4**	**1844.4**

PARAMETERS
CALCULATED PARAMETER
CALCULATED COST
MODIFIED (from scenario 1 in main)
